# Effectiveness of Interventions to Modulate the Rumen Microbiota Composition and Function in Pre-ruminant and Ruminant Lambs

**DOI:** 10.3389/fmicb.2018.01273

**Published:** 2018-06-18

**Authors:** Cristina Saro, Ulli M. Hohenester, Mickael Bernard, Marie Lagrée, Cécile Martin, Michel Doreau, Hamid Boudra, Milka Popova, Diego P. Morgavi

**Affiliations:** ^1^Université Clermont Auvergne, INRA, VetAgro Sup, UMR Herbivores, Saint-Genès-Champanelle, France; ^2^Herbipôle, INRA, Saint-Genès-Champanelle, France; ^3^Université Clermont Auvergne – CNRS – SIGMA-Clermont, Institut de Chimie de Clermont-Ferrand, Clermont-Ferrand, France; ^4^Université Clermont Auvergne – INRA, MetaboHUB/Plateforme d’Exploration du Métabolisme, Clermont-Ferrand, France

**Keywords:** rumen, lambs, early life, microbial colonization, methane, next-generation sequencing, metabolomics

## Abstract

Modulating the assembly of the ruminal microbiota might have practical implications in production. We tested how an early-life dietary intervention in lambs influences the diversity and function of the ruminal microbiota during and after the intervention. Microbiota resilience during a repeated dietary intervention was also tested. The treatment, aiming to mitigate enteric methane emissions, combined garlic essential oil and linseed oil. Fifty-six lambs and their dams were allocated to two groups and treatment (T1) or placebo (C1) was drenched from birth until 10 weeks of life. Lambs were weaned at 8 weeks. From 16 to 20 weeks, lambs in each group were divided in two subgroups that received (T1–T2 and C1–T2) or not (T1–C2 and C1–C2) the same treatment. Measurements were done at 8, 14, and 20 weeks. Average daily gain was similar between groups. Methane production was reduced by treatment at 8 and 20 weeks but at 14 weeks it was similar between C1 and T1. Interestingly, early-life treated lambs displayed a numerical increase (*P* = 0.12) in methane emissions at 20 weeks compared with non-treated lambs. Concentration of VFA was not affected by the intervention at 8 or 14 weeks but a lower concentration was observed in T2 lambs compared with C2 at week 20. Metataxonomics (rRNA gene) revealed differences in archaeal communities between groups of lambs when treatment was applied (weeks 8 and 20); whereas, in accord with methane emissions, these differences disappeared when treatment was discontinued (week 14). Protozoal community structure was not affected by treatment. In contrast, bacterial community structure differed between treated and non-treated lambs during and after the intervention. Rumen and urine LC-MS and NMR metabolomics at week 20 separated C2 from T2 lambs and correlation analysis highlighted interactions between microbes and metabolites, notably that of methylated compounds and Methanomassiliicocceae methanogens. This study demonstrates that a long-term early-life intervention induced modifications in the composition of the rumen bacterial community that persisted after the intervention ceased with little or no effect on archaeal and protozoal communities. However, there was no persistency of the early-life intervention on methanogenesis indicating resilience for this function.

## Introduction

The colonization of the gastrointestinal track of newborns starts during birth and continues through successive waves of colonization and community changes until the microbiota reaches a stable state later in life. The duration of this succession of symbiotic microbes up to when the microbiota attains a relative stability will depend on the animal species and diet. In ruminants, the development of the rumen and feeding on solid feeds are determinants. Microbes colonize rapidly the rumen after birth in a defined and progressive way (Fonty et al., 1987, 1988; Jami et al., 2013; Rey et al., 2014), a process that is affected by the surrounding environment such as diet and the contact with congeners. Changes in early succession of primary colonizers can affect the composition and function of the community in mature animals (Morgavi et al., 2015). In addition, it has been proven that early-life experiences might have long-standing health consequences (Gensollen et al., 2016).

Various events or disturbances [as defined by Shade et al. (2012)] may directly or indirectly modify the gastrointestinal microbiota composition. These modifications may be particularly significant early in life, during the period of successive colonization when the microbiota is less stable and comparatively simple with few dominant taxa. In contrast, a diverse and mature microbial community, such as the observed in the rumen of adult ruminants, is both more resistant to disturbances and have higher resilience than less mature communities (Lozupone et al., 2012; Shade et al., 2012). Consequently, in adult ruminants the composition and function of the microbiota generally return to pre-intervention values when the disturbance is removed. Whereas, in young ruminants, there is some evidence that effects of disturbance could persist for some time after the end of the intervention. Yáñez-Ruiz et al. (2010) showed that rumen bacterial communities differed in two groups of 9-month-old lambs fed the same diet but that were fed different diets during the first 4 months of life. In contrast, no differences were observed in the archaeal community and methane emissions. Abecia et al. (2013) performed an experiment with goat kids treated or not with a methanogen inhibitor from birth until 1 month after weaning. The dams were treated or not, forming four groups. Treated kids remained lower emitters than non-treated kids 4 months after the end of the treatment but this persistent effect was observed only in kids raised by treated dams. Finally, Lyons et al. (2017) treated lambs with linseed oil from birth to 6 weeks of age and studied the bacterial and archaeal communities 2 months after the end of the treatment. They reported differences in the bacterial community of the two groups of lambs. On the other hand, no differences were found in the archaeal community or volatile fatty acids (VFA) profile between treated and non-treated lambs. These results are encouraging; however, more research is necessary to gain a better understanding of the rumen colonization process and the way it can be modulated. An aspect that have been less studied is how microbial communities that were affected by disturbance events during their *de novo* assembly in young animals will react to the same type of disturbance later in life. Whether these communities will be more or less resilient is an important question in microbial ecology that has practical implications. For instance, the animal response to a dietary treatment could be different for phenotypes driven by the gut microbiota such as methane emissions and forage digestibility depending on whether or not the microbial community was previously exposed.

The aim of this study was (a) to monitor the changes occurring in the composition and function of the rumen microbiota of lambs when a dietary treatment (disturbance) was applied from birth up to 10 weeks, (b) to check if those changes persisted after the end of the treatment, and (c) to check the possible influence of the early experience on the response to the treatment reapplied later in life. In this study methanogenesis and fermentation patterns were the microbial functions that we used to monitor the effect of ecosystem disturbance induced by a dietary treatment. The treatment chosen was a combination of garlic essential oil and linseed oil. Both compounds have been shown to be effective for reducing methane emissions (Macheboeuf et al., 2006; Mateos et al., 2013; Guyader et al., 2015; Patra and Yu, 2015) and were combined as they have different mechanisms of action. Organosulfur compounds contained in garlic inhibits methanogens at low concentrations (Jouany and Morgavi, 2007), whereas lipids have a broader inhibitory effect on microbes, particularly protozoa and some bacteria that have an effect on H_2_ production (Soliva et al., 2004; Guyader et al., 2014). Newborn lambs were separated in two groups that were or not treated and the effects of the treatment were checked at the end of the administration and 1 month later. After that, lambs were separated into four groups and the treatment was reapplied to half of the previously control lambs and half of the previously treated lambs with the effect of the first and second treatment evaluated at the end of this second administration.

## Materials and Methods

Procedures with animals were conducted in accordance with the guidelines for animal research of the French Ministry of Agriculture and applicable European guidelines and regulations for experimentation with animals (French Research Ministry approval number: 9454-2017033017248737).

### Animals, Diets and Treatment

**FIGURE 1:**
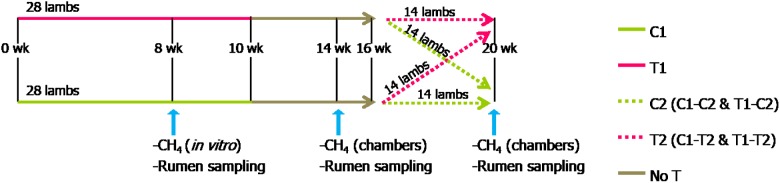
Experimental design and sampling schedule for control (C) and treated (T) lambs from birth to 10 weeks of age (C1 and T1) and from 16 to 20 weeks of age (C2 and T2). Blue arrows indicate sampling and methane measurements. No treatment was applied from weeks 10 to 16 (No T).

Thirty gestating ewes were used in this study. Ewes carrying twins were recruited the day of lambing within a 7-day period (only one ewe gave birth 3 days before). Immediately after delivery, ewes and lambs were allocated into two groups equilibrated for male:female ratio of lambs. The allocation parameters used were lambs’ day of birth, weight at birth and sibling (male/male, female/female, female/male). Each group was housed in the same barn but in separate pens with no contact between animals of different groups. Ewes in both groups received a diet for lactating sheep (hay *ad libitum*, 200 g dehydrated alfalfa, 420 g barley per day). Lambs had free access to a natural grassland hay [921.1 g dry matter (DM)/kg fresh matter (FM), 899.7 g organic matter (OM)/kg DM, 535.9 g neutral detergent fiber (NDF)/kg DM, 110.0 g protein/kg DM] and concentrate (889.0 g DM/kg FM, 935.0 g OM/kg DM, 87 g NDF/kg MS, 170 g protein/kg DM). Two lambs per group died of natural causes in the first few days after birth. Lambs remained with their dams until weaning that was done when the youngest lamb was 8 weeks old. Water was freely available through waterers. From weaning, lambs stayed in the same pen and received a typical ration for growing lambs with natural grassland hay *ad libitum* and a fixed amount of concentrate that was decreased progressively from 1000 g/lamb/day at weaning until 500 g/lamb/day at week 11. One group received a treatment (T1) consisting in linseed oil (1.6 mL/kg BW) and garlic essential oil (3 μL/kg BW, a gift from Phytosynthese, Mozac, France) and the other received the same volume of beet molasses (control). Treatment was administered once daily with a drencher until 4 weeks of age and every other day from week 5 until 10 weeks of age. The alternate day administration was done because a few lambs developed a mild aversion to the treatment. Ewes in the treated and control groups received the treatment or the placebo, respectively, mixed with the concentrate. Weight of lambs was weekly recorded. At 16 weeks of life, lambs in both groups were subdivided into two groups that received (T1–T2 and C1–T2) or not (T1–C2 and C1–C2) the same treatment during four additional week. The experimental plan and sampling are shown in **Figure 1**.

### Methane Measurements

Methane measurements were done after weaning at 8, 14, and 20 weeks of age. At 8 week, methane emission potential was determined *ex vivo* using rumen fluid obtained from lambs via esophageal tubing. Rumen fluid was kept warm and immediately transferred to the laboratory. Two mL of rumen fluid were mixed with 3 mL of buffer solution (Goering and Van Soest, 1970) in a Hungate tube under anaerobic conditions and incubated for 24 h at 39°C in order to determine methane production potential. At week 14 and 20 of life (4 weeks after the end of T1 and at the end of T2) methane emissions from lambs were measured in open circuit respiration chambers constructed of metal frame and polycarbonate. Due to the small size of lambs, 2–3 lambs were simultaneously measured in each chamber. Each chamber measured 1.6 m wide × 2.05 m deep × 2.00 m high and had a volume of 6.6 m^3^. Chambers were opened once a day for less than 10 min in order to clean the floor and feed the animals.

Concentration of gasses in the barn and in the four chambers was alternatively analyzed at a 0.1-Hz sample frequency for 5 min every 25 min using an infrared detector (Ultramat 6; Siemens, Karlsruhe, Germany) and recorded (Nanodac Invensys recorder/controller; Eurotherm Automation SAS, Dardilly, France). The detector was manually calibrated the day before each measurement period using pure N_2_ and a mixture of CH_4_ (650 mg/kg) and CO_2_ (700 mg/kg) in N_2_. Chamber doors were never opened during gas analysis, so no data was deleted. Real-time gas emissions in a chamber were calculated by the difference between chamber and ambient gas concentrations multiplied by the airflow (Pinares-Patino et al., 2012). Measurements were done during 3 days and daily methane emissions were averaged.

### Sampling

After weaning, at 8, 14, and 20 weeks of life rumen fluid was sampled via esophageal tubing before morning feeding. One mL was stored at -80°C until DNA extraction and 0.8 mL were mixed with 0.5 mL of deproteinizing solution (4 g of crotonic and 20 g of metaphosphoric acid/L in 0.5 N ClH) for VFA analysis. In addition, at 20 weeks 500 μL of rumen fluid were immediately stored at -80°C until metabolome analysis. Urine samples were collected at 20 weeks for metabolome analysis. Collection differed between female and male lambs. At the end of the study females were kept in the herd for reproduction and males were slaughtered. Urine samples (500 μL) from slaughtered males were taken directly from the bladder at the abattoir whereas females were stimulated to urinate. Samples were immediately stored at -80°C until analysis.

### Microbial Community Analysis

DNA was extracted according to the method developed by Griffiths et al. (2000), quantified by fluorometry using a Qubit (Thermo Fisher Scientific, France) and run on a FlashGel System (Lonza, Rockland, Inc., France) to check integrity. Rumen gDNA was submitted to the Roy J. Carver Biotechnology Center (Urbana, IL61801, United States) for DNA library preparation using Fluidigm amplification and sequencing using the Illumina HiSeq platform. Selected primers for archaeal and bacterial 16S rRNA and protozoal 18S rRNA are described in Supplementary Table S1.

The sequence data have been submitted to the NCBI BioProject database under ID PRJNA438821. Analysis of the sequences were made independently for each primer pair. Approximately 15,000,000 raw archaeal sequences were generated. Reads were assigned to their respective samples and trimmed of barcodes. Reads were merged using the make.contig instruction from mothur (Schloss et al., 2009) and resulting sequences were subjected to a quality filter (mean phred score ≥ 25, length ≥ 250, maximum five primer mismatches, <8 homopolymers). Operational taxonomical units (OTUs) were clustered at 97% of similarity using QIIME (Caporaso et al., 2010b). Sequences were aligned using Pynast (Caporaso et al., 2010a) and checked for chimera using the chimera.uchime option in mothur. Sequences marked as chimeras were removed and taxonomy was assigned against the RIM-DB database (Seedorf et al., 2014).

Raw bacterial and protozoal reads (approximately 18,000,000 and 8,000,000, respectively) were analyzed using the IM-Tornado pipeline (Jeraldo et al., 2014), a tool designed to analyze sequencing data producing two separate reads that do not overlap. Silva database release 123_1 was used in both cases to assign taxonomy.

Finally, abundance of archaea and bacteria were assessed by qPCR amplifying *mcrA* gene in archaeal community and 16S rRNA bacterial gene. Primers used are listed in Supplementary Table S1.

### Sample Analysis by LC-MS and NMR

Metabolite profiling of rumen fluid and urine samples were performed on a Metabolic Profiler system (Bruker, Wissembourg, France) which combines a liquid chromatography (Agilent 1200 Series Fast LC System) coupled with a MicrOTOF mass spectrometer (Bruker Daltonics, Bremen, Germany) and a NMR instrument (Avance III 500MHz, Bruker Biospin GmbH, Rheinstetten). To ensure the LC-MS system’s reproducibility and stability, six quality control samples (QCs) were injected three times, at the beginning, middle, and the end of the run.

### Data Processing and Statistical Analysis

Data from methane production, VFA concentration, qPCR abundance and alpha diversity indexes were analyzed independently for each time point using the PROC MIXED procedure in SAS 9.4 (SAS Institute Inc., Cary, NC, United States). The fixed factor was the treatment (for 8 and 14 weeks) or both treatments and their interaction (20 weeks) and animal was the random factor. For methane production at weeks 14 and 20, the experimental unit was the chamber. Data of relative abundances of different microbial communities were analyzed using the non-parametric Kruskal–Wallis test and the Benjamini–Hochberg correction using R (R Foundation for Statistical Computing^1^). Non-metric multidimensional scaling (NMDS) plots were constructed using OTU-based Bray–Curtis dissimilarities distance in the R package ggplots2 (Wickham, 2009) and the dist and metaMDS functions of the R package vegan (Oksanen et al., 2016). Permanova analysis to compare groups in the PCoA plots was performed with the adonis function in the R package vegan. Betadisper and simper functions from the same package were used to assess the differences in dispersions between groups and the contribution to variability of the different microbial groups.

Mass spectrometry data were processed using the XCMS software package included in a Galaxy workflow (Giacomoni et al., 2015). Filtration and normalization steps were additionally applied. First, signals outside the analytical interesting range of retention time from 0.4 to 22 min were removed from the datasets. The filtered features were then normalized, the batch effects and signal drifts were fitted by a linear regression model to the QC values (van der Kloet et al., 2009). For NMR data, acquired noesygppr1d 1H-NMR spectra were transferred into a bucket table (Spraul et al., 1994) with 0.01 ppm bucket size. The generated feature tables for both MS and NMR data were analyzed by principal component analysis (PCA) and orthogonal projections to latent structures-discriminant analysis (OPLS-DA) using SIMCA-P software (Umetrics, Umeå, Sweden). PCA was first used to look for trends and/or outliers. No clear separation was obtained for both MS and NMR data (Supplementary Figure S1). Supervised OPLS-DA models were used to reveal potential markers of response to treatment. The model for MS data showed a clear separation with acceptable predictive parameter (*Q*^2^ = 0.751 and 0.634 for rumen fluid and urine, respectively), and its validation was performed by permutation tests with 200 permutations and the CV-score. For NMR data, a multivariate variable selection was necessary to improve the predictive ability of OPLS-DA models (*Q*^2^ = 0.809 and 0.398 for rumen fluid and urine, respectively) as unfiltered NMR data had low *Q*^2^ values. Because each molecule have several signals in the NMR spectra and each signal is sliced into several buckets, the VIP were combined to an average VIP. Ion species, corresponding to the features with VIP’s above 1 were selected for annotation. For LC-MS metabolites, identification experiments were performed on an UltraMate 3000 (Dionex, Les Ulis, France), coupled to an Orbitrap Velos (Thermo, Les Ulis, France), using the same LC conditions as for the LC-MS acquisitions used before (see Supplementary Note 1 for details). Identification of all discriminant metabolites was confirmed by comparison of their exact mass, retention time and fragmentation spectra with those obtained using standards. For NMR metabolites, identification was done by two-dimensional (2D) NMR experiments (Noesygppr1d, JRes, cosygpprqf, Jresgpprqf, dipsi2phpr, HSQCetgpsi, and HMBCgplpndqf) at 298 K. Semi-quantitative metabolite concentration was calculated using the geometric average of the signal intensities and compared between control and treated samples.

## Results

Average daily gain, measured at 14 and 20 weeks of age was not different between control and treated lambs (0.212 kg/lamb/day; *P* > 0.05).

### Effect of Treatment on Methane Production and Rumen Fermentation Functions

The treatment reduced by 23% the methane production potential in 8-week-old lambs (*P* = 0.0046, **Table 1**). In contrast, no differences were found in methane production or methane yield (*P* > 0.05) at 14 weeks of life, i.e., 1 month after the end of the treatment (T1). At 20 weeks of life, at the end of the second treatment period (T2), methane emissions were lowered by 14% in treated compared to control lambs (*P* = 0.0532 for production and *P* = 0.0192 for yield). Although no significant interaction was detected between T1 and T2, there were numerical differences among the four groups with consistently higher methane emissions observed in lambs treated early in life. The biggest difference at 22% was observed between C1–T2 and T1–C2.

**Table 1:** Values of methane production potential (8 weeks), methane emissions and methane yields measured in respiration chambers (14 weeks) of control lambs (C1) and lambs treated with a linseed-garlic combination (T1) from birth up to 10 weeks of age.

	C1		T1		*SEM*	*P*-value		
	
						T1	T2	T1 × T2
8 weeks								
Methane (*in vitro*; μmol/mL)^∗^	7.30		5.65		0.393	0.0046	n/a	n/a
14 weeks								
Methane (g/day)^∗^	20.27		21.30		1.398	0.6093	n/a	n/a
Methane yield (g/kg DM intake)^∗^	19.68		19.98		1.032	0.8357	n/a	n/a
	
	**C1–C2**	**C1–T2**	**T1–C2**	**T1–T2**				
	
20 weeks								
Methane (g/day)^∗^	25.65	21.64	27.81	24.25	1.844	0.2109	0.0532	0.9041
Methane yield (g/kg DM intake)^∗^	18.57	15.81	20.17	17.58	1.051	0.1252	0.0192	0.9401

*At 16 weeks of age four groups were formed and lambs were retreated or not with the same treatment for 4 weeks (C2 and T2, 20-week measures).*

*^∗^n = 52 for 8 weeks in vitro methane determination, 20 for 14 weeks in vivo methane determination and 24 for 20 weeks in vivo methane determination. n/a not applicable.*

Total VFA concentration and VFA profiles are presented in **Table 2**. Whereas no differences were induced by the treatment in 8-week-old and 14-week-old lambs (*P* > 0.05), a decrease in total VFA concentration was detected at 20 weeks in T2 lambs (C1–T2 and T1–T2) when compared with C2 lambs (C1–C2 and T1–C2; *P* = 0.0015). Similar to methane emissions, the biggest differences were observed between C1–T2 and T1–C2 lambs. The VFA profile at 20 weeks was also affected by treatment with lower molar proportions of acetate and higher molar proportions of propionate in T2 lambs. Acetate to propionate ratio was consequently reduced by treatment (*P* = 0.0014).

**Table 2:** Values of total volatile fatty acids (VFA) concentration, proportions of acetate, propionate, butyrate and other VFA, acetate to propionate ratio in the rumen and average daily gain of control lambs (C1) and lambs treated with a linseed-garlic combination (T1) from birth up to 10 weeks of age.

	C1		T1		*SEM*	*P*-value		
	
						T1	T2	T1 × T2
8 weeks							
Total VFA (mM)	28.49		24.60		2.150	0.9260	n/a	n/a
% Acetate	72.40		74.14		0.865	0.1614	n/a	n/a
% Propionate	18.13		16.89		0.851	0.3094	n/a	n/a
% Butyrate	5.33		4.87		0.3177	0.3145	n/a	n/a
% Others^∗^	4.41		4.10		0.495	0.9486	n/a	n/a
Ac:Pr	4.38		4.78		0.312	0.2962	n/a	n/a
14 weeks							
Total VFA (mM)	46.36		40.39		2.816	0.7965	n/a	n/a
% Acetate	71.51		71.25		0.627	0.7788	n/a	n/a
% Propionate	15.69		16.10		0.465	0.5340	n/a	n/a
% Butyrate	7.34		7.43		0.333	0.8439	n/a	n/a
% Others^∗^	5.47		5.21		0.355	0.5953	n/a	n/a
Ac:Pr	4.64		4.59		0.177	0.8482	n/a	n/a
ADG (kg/day)	0.283		0.283		0.009	0.9530	n/a	n/a
	
	**C1–C2**	**C1–T2**	**T1–C2**	**T1–T2**				
	
20 weeks							
Total VFA (mM)	53.67	40.16	58.19	45.67	3.878	0.2021	0.0015	0.8992
% Acetate	70.05	67.59	68.73	67.23	0.714	0.2446	0.0079	0.5038
% Propionate	14.05	15.94	15.14	15.96	0.551	0.3169	0.0175	0.3379
% Butyrate	9.37	9.18	10.22	9.62	0.346	0.0695	0.2602	0.5591
% Others^∗^	6.53	7.29	5.91	7.19	0.449	0.4293	0.0278	0.5674
Ac:Pr	5.01	4.30	4.66	4.29	0.160	0.2608	0.0014	0.3024
ADG (kg/day)	0.253	0.251	0.258	0.251	0.011	0.8082	0.7003	0.7922

*At 16 weeks of age four groups were formed and lambs were retreated or not with the same treatment for 4 weeks (C2 and T2).*

*n = 28 for C1, 28 for T1 8 weeks, 26 for T1 14 weeks, 14 for C1–C2, C1–T2, T1–C2, 13 for T1–T2. ^∗^Other VFA represent the sum of isobutyrate, isovalerate, valerate, and caproate. n/a not applicable.*

### Rumen Methanogens Community Was Altered by Treatment but Recovered After Treatment Stopped

A total of 5,481,453 archaeal reads were retained for the analysis of the archaeal community, with 33,220 ± 15,833 reads per sample (mean ± SD). A total of 185 archaeal OTUs were represented across all samples. Alpha diversity was evaluated with the Chao estimator, the number of observed OTU and the Shannon diversity index (**Table 3**). All indices were reduced by T1 at 8 and 14 weeks and by T2 at 20 weeks. However, at 20 weeks of age, the number of archaeal OTU was higher in lambs that had been previously treated (T1) than in non-treated (C1) lambs, and the Chao estimator tended to be higher in those lambs. In addition, alpha diversity indices increased as lambs aged.

**Table 3:** Alpha diversity of archaeal community (Chao 1, observed OTU and Shannon index) in the rumen of control lambs (C1) and lambs treated with a linseed-garlic combination (T1) from birth up to 10 weeks of age.

Archaea	C1		T1		*SEM*	*P*-value		
	
						T1	T2	T1 × T2
8 weeks						
Chao1	116.74		79.59		8.695	0.0039	n/a	n/a
Observed OTU	101.93		63.79		8.049	<0.0001	n/a	n/a
Shannon index	2.83		2.04		0.159	0.0009	n/a	n/a
14 weeks								
Chao1	157.48		130.66		7.730	0.0185	n/a	n/a
Observed OTU	145.36		117.96		7.658	0.0145	n/a	n/a
Shannon index	3.21		2.77		0.147	0.0417	n/a	n/a
	
	**C1–C2**	**C1–T2**	**T1–C2**	**T1–T2**				
	
20 weeks						
Chao1	177.45	151.49	179.19	175.95	7.031	0.0685	0.0432	0.1129
Observed OTU	159.64	138.79	169.85	160.08	7.408	0.0386	0.0441	0.4581
Shannon index	3.57	3.04	3.60	3.11	0.153	0.7444	0.0017	0.8984

*At 16 weeks of age four groups were formed and lambs were retreated or not with the same treatment for 4 weeks (C2 and T2).*

*n = 28 for C1, 28 for T1 8 weeks, 26 for T1 14 weeks, 14 for C1–C2, C1–T2, T1–C2, 13 for T1–T2. n/a not applicable.*

Relative abundance was calculated for archaeal clades grouped according to the classification of Janssen and Kirs (2008) (Supplementary Table S2). The *Methanobrevibacter gottschalskii* clade was the most abundant regardless of age and treatment. At 8 weeks, T1 lambs had a higher abundance of *M. boviskoreanii/wolinii* clade and a lower abundance of *M. ruminantium* clade (*P* < 0.05) than C1 lambs. In addition, the abundance of Methanomassiliicoccales decreased and that of *Methanosphaera* increased in treated lambs, although the effect was not significant. At 14 weeks of age, no difference in the relative abundance of any archaeal clade was observed between the two groups. At 20 weeks of age, the linseed oil-garlic treatment affected the relative abundance of Methanomassiliicoccales (*P* < 0.05) in C1–T2 lambs compared with T1–C2 lambs. When comparing groups using the Bray–Curtis dissimilarity matrix and the adonis function for the three time points, the test revealed a separation between T1 and C1 groups in week 8 and between T2 and C2 groups in week 20, whereas no clear grouping pattern was observed in week 14 (*P* > 0.05). No difference was observed between T1 and C1 lambs at 20 week according to the adonis test.

**FIGURE 2:**
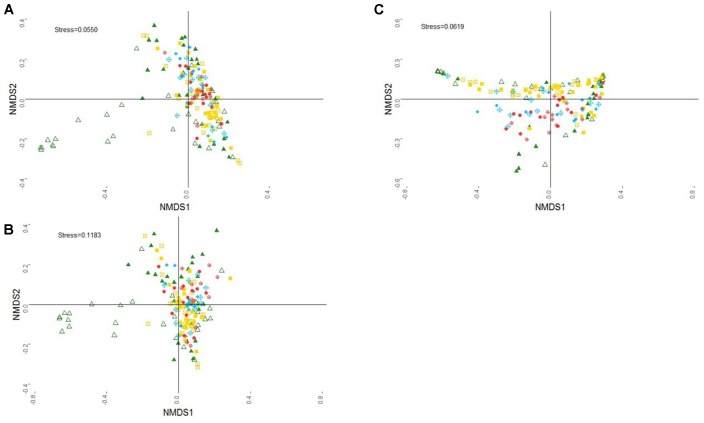
Non-metric multidimensional scaling (NMDS) plot of rumen **(A)** archaeal, **(B)** bacterial, and **(C)** protozoal community structure. All samples across week are represented: 8 week-C (green shaded triangle), 8 week-T (green open triangle), 14 week-C (yellow shaded square), 14 week-T (yellow open square), 20 week-C1–C2 (red shaded circle), 20 week-T1–C2 (red open circle), 20 week-C1–T2 (blue shaded diamond), 20 wk-T1–T2 (blue open diamond).

When all samples across the three time points are represented together in a NMDS plot (**Figure 2A**), it is observed that samples from older animals grouped closer together and that at 8 and 14 weeks of age samples from treated lambs were more scattered than those from control lambs suggesting that the intervention affected the maturation of the community.

Finally, when assessing archaeal abundance using qPCR and the *mcrA* gene, we observed no difference between C1 and T1 lambs at 8 weeks (7.67 vs. 7.61 log_10_ copies/mL rumen content, respectively; *P* > 0.05). When checking the difference at 14 weeks, abundance of methanogens was higher in C1 group (5.99 vs. 5.46 log_10_ copies/mL rumen content; *P* = 0.0005). This difference still persisted at 20 weeks, 6.10 vs. 5.74 log_10_ copies/mL rumen content for C1 and T1, respectively (*P* = 0.0071). However, when comparing lambs that had been treated for the second time, no differences were found in the abundance of methanogens (5.87 in T2 vs. 5.97 in C2 log_10_ copies/mL rumen content; *P* > 0.05).

### Bacterial Communities Were Modified During and After Treatment but Protozoa Remained Unchanged

A total of 6,063,537 bacterial sequences were retained for analysis, with 39,120 ± 26,943 reads per sample (mean ± SD). There was no difference in alpha diversity indices between treated and control lambs at 8 and 14 weeks but T2 reduced diversity at 20 weeks of age (Chao 1 estimator 978.8 vs. 891.2, observed OTU 843.9 vs. 758.5 and Shannon index 6.93 vs. 6.54 for control and treated lambs, respectively; *P* < 0.05).

Treatment influenced the relative abundance of major phyla Firmicutes and Bacteroidetes (Supplementary Table S3). At 8 and 14 weeks the Firmicutes increased (*P* < 0.05) in treated animals whereas Bacteroidetes decreased (*P* < 0.05 at 14 weeks, numerical decrease at 8 weeks). However, at 20 weeks there was a shift in proportions as treated lambs had higher relative abundance of Bacteroidetes and lower relative abundance of Firmicutes than controls. Some phyla were consistently modified by T1 and T2 like Synergistetes and Cyanobacteria that increased (*P* < 0.05) and Fibrobacteres whose relative abundance decreased by 2/3 of that of control (*P* = 0.0007 at 8 weeks and *P* = 0.0004 at 20 weeks). No differences between groups were observed at 14 weeks for those phyla.

For a better insight of the effects of treatment on bacteria, we analyzed differences at a lower taxonomical level. We selected families and bacterial groups at family level that had an abundance higher than 0.1% across samples (Supplementary Table S4). At 8 weeks of life, the relative abundance of Fibrobacteriaceae, Rikenellaceae, Clostridiales vadinBB60 group, Bacteroidetes VC2 1 Bac22 and Anaeroplasmataceae was higher in the control group, whereas that of Lachnospiraceae, Ruminococcaceae, and Veillonellaceae was higher in the treated group. Simper analysis indicated that the most influential bacterial families, accounting for 52% of the variability between treated and untreated lambs, were Prevotellaceae, Fibrobacteriaceae, Christensenellaceae, and Lachnospiraceae. Also, the bacterial community structure at 8 weeks differed between control and treated lambs (adonis *P* = 0.005). At 14 weeks, Prevotellaceae and Rhodospirillaceae were more abundant in the control group and Lachnospiraceae, Ruminococcaceae, Bacteroidetes VC2 1 Bac22, Spirochaetaceae, Acidaminococcaceae, Anaerolineaceae, and Anaeroplasmataceae were more abundant in the treated group. Prevotellaceae, Christensenellaceae, Rikenellaceae, and Lachnospiraceae were the most influential bacterial families, accounting for 52% of the contribution to variability (simper analysis). Interestingly, the bacterial community structure was still different between control and treated animals (adonis *P* = 0.001) at 14 weeks of age, 4 weeks after the treatment was stopped. When analyzing the differences in bacterial families between the four groups at 20 weeks, significant differences were found in 12 families as shown in Supplementary Table S4. Prevotellaceae was the most abundant family, and its abundance was higher in all the groups that were treated, either in T1 or T2, compared to animals that never received the treatment (group C1–C2). Similarly, Lachnospiraceae abundance was lower in the three treated groups than in the C1–C2 group but T1 treatment had the opposite effect at 8 and 14 weeks of age. Fibrobacteriaceae and Bacteroidales RF16 group were significantly reduced in T2 animals compared to C2 animals, regardless the treatment received in the first period. When performing the simper analysis to check the species influencing the variability between groups according to the second treatment, Prevotellaceae, Christensenellaceae, Lachnospiraceae, Rikenellaceae, and Bacteroidales S24.7 group accounted for 59% of the variability. Finally, when performing the adonis analysis, bacterial communities had a different structure whether they were analyzed according to T1 (*P* = 0.039) or T2 (*P* = 0.004). When all samples were represented in a unique NMDS plot (**Figure 2B**), a similar tendency than that observed with the archaeal community was observed. Samples from 8- and 14-week-old lambs were more scattered than those from week 20, supporting the hypothesis that as the animal ages, the microbial community within individuals in a group matures and develops in a convergent way. When analyzing the dispersion of the groups (8, 14, and 20 weeks), betadisper function showed a significant difference between the dispersion of the group at 20 weeks and the dispersions at 8 or 14 weeks. Also, at weeks 8 and 14, samples from treated animals appeared to be more scattered on the plot than samples from control animals, which, at 14 weeks could indicate a delay in maturation of bacterial community in animals that had been previously treated.

Bacterial abundance was assessed by qPCR using the 16S rRNA gene. No differences were observed between C1 and T1 animals at 8 weeks (10.04 vs. 10.06 log_10_ copies/mL rumen content for C1 and T1; *P* > 0.05). At 14 weeks, abundance was higher in C1 group (9.92 vs. 9.61 log_10_ copies/mL rumen content; *P* = 0.0316). This difference persisted at 20 weeks, with lower bacterial abundance in T1 lambs than in C1 lambs (6.10 vs. 5.74 log_10_ copies/mL rumen content; *P* = 0.0071). Similarly to methanogens, there were no differences between T2 and C2 (9.78 vs. 9.75 log_10_ copies/mL rumen content; *P* > 0.05).

The number of reads retained for the protozoal sequences analysis was 5,713,957 with an average of 36,394 ± 18,574 reads per sample (mean ± SD). Shannon diversity index tended to be lower in T1 lambs at 8 weeks (*P* = 0.0539) and T2 lambs at 20 weeks (*P* = 0.0554) whereas no differences were observed at 14 weeks. No differences were observed in Chao 1 estimator or number of observed OTU for any of the time points. Seven protozoal genera were identified: *Dasytricha, Enoploplastron, Entodinium, Eremoplastron, Isotricha, Ophryoscolex*, and *Polyplastron*. Relative abundances are shown in Supplementary Table S5. At 8 weeks of life, relative abundance of Ophryoscolex was lower in control lambs, whereas that of *Polyplastron* (both *Polyplastron* sp. and *Polyplastron* sp. LDK 2011) was lower in treated animals. At 14 weeks of age, differences were observed in *Dasytricha* and *Isotricha*, which were both less abundant in treated animals. Finally, at 20 weeks differences in relative abundance were observed in *Enoploplastron* *triloricatum* and *Eremoplastron* (both higher in T1–C2 animals). Adonis function revealed a difference in the protozoal community at 8 weeks between T1 and C1 groups. The genera that contributed most to the variability were *Entodinium* and *Ophryoscolex* (71%) according to the simper analysis. No difference in the structure of both groups was observed at 14 weeks (adonis: *P* > 0.05) and in this case the groups that contributed most to the variability were also *Ophryoscolex* and *Entodinium* (71%). Finally, at 20 weeks adonis revealed a structural difference between communities when they were analyzed according to T2 but no difference was detected when the groups were compared according to T1. *Entodinium* and *Ophryoscolex* were again the genera that contributed most to the variability (55%) when analysis was performed according to T2.

As for bacteria and archaea, the NMDS plot shows a larger scattering for samples at 8 weeks than samples at 14 and 20 weeks (confirmed by the betadisper test, *P* < 0.05). However, control and treated animals were not clearly separated (**Figure 2C**).

### Changes in Rumen and Urine Metabolome Associated to Treatment and Microbial Groups

**FIGURE 3:**
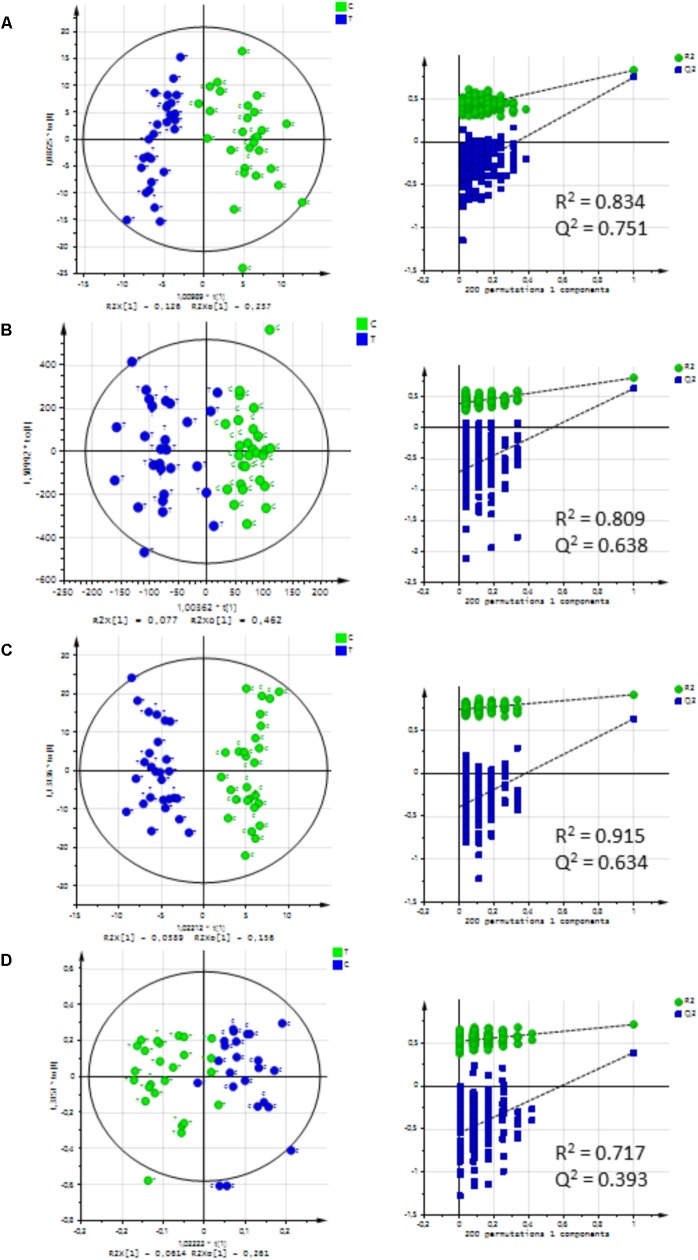
Orthogonal projections to latent structures-discriminant analysis (OPLS-DA) and validation permutation tests of rumen fluid and urine samples from control (C, *n* = 28 rumen fluid, *n* = 27 urine) and treated (T, *n* = 27) lambs at 20 weeks of age. **(A)** Rumen fluid analyzed by LC-MS (284 variables). **(B)** Rumen fluid analyzed by NMR after variable selection (582 of 641 variables). **(C)** Urine analyzed by LC-MS (848 variables). **(D)** Urine analyzed by NMR after variable selection (939 of 1030 variables).

Metabolic profiles in rumen fluid and urine differed between treated and untreated lambs at 20 weeks of age (**Figure 3** and Supplementary Figure S1). Discriminant metabolites are shown in **Table 4**. A group of seven metabolites having the highest VIP for the LC-MS method could be structurally characterized (M8 to M14; Supplementary Figure S2) based on their fragmentation pattern but they have not been described before and their functions are unknown. Due to their structural similarity, it is expected that they belong to a related pathway(s). Other discriminant metabolites were choline that decreased in T2 lambs whereas 3-deoxycarnitine (also named 4-trimethylammoniobutanoic acid) and pipecolic acid increased in this group. The NMR analysis resulted in the annotation of eight metabolites, mostly carboxylic acids, with VIP ≥ 0.9. In treated lambs, acetic acid and butyric acid decreased whereas isobutyric acid, alanine, malic acid, phenylacetic acid, p-hydroxyphenylacetic acid, and suberic acid increased (**Table 4**). In urine, up to 16 metabolites with VIP ≥ 1 were identified, notably trimethylamine and related metabolites such as choline and betaine that have been associated with methylotrophic methanogens (**Table 4**). Identification of discriminant metabolites in urine by LC-MS was challenging due to the different sampling conditions and the large variability in sample osmolarity that ranged from 226 to 1586 mOsm/L (Supplementary Figure S3). In addition, only few VIPs had signal intensities strong enough for annotation by fragmentation experiments. Notwithstanding, taurine and trimethylamino-oxide (TMAO) could be annotated and all decreased in treated samples.

**Table 4:** Changes in rumen and urine metabolites detected by LC-MS/ToF and NMR between control and treated lambs at 20 weeks.

Metabolite	Matrix	VIP	Change^‡^	*P*-value	*t*_R_ (min)	[M+H]^+^ exptl.	Δm/z in ppm
LC-MS						
M12	Rumen	2.1	↘	<0.001	3.4	215.10263	-0.9
M13	Rumen	2.1	↘	<0.001	3.9	229.11750	-3.4
M10	Rumen	1.9	↘	<0.001	2.9	199.10883	5.6
M9	Rumen	1.9	↗	<0.001	2.5	185.09282	4.0
M14	Rumen	1.8	↗	<0.001	5.2	245.11298	-0.9
M8	Rumen	1.7	↘	<0.001	2.2	169.09685	-1.8
M11	Rumen	1.7	↘	<0.001	11	201.12390	2.6
Choline	Rumen	1.0	↘	<0.001	18.8	104.10701	0.2
3-Deoxycarnitine^†^	Rumen	1.0	↗	<0.001	18.2	146.11749	3.9
Pipecolic acid	Rumen	1.0	↗	<0.001	13.2	130.08677	-0.4
	
Trimethylamine N-oxide	Urine	1.4	↘	<0.001	16.6	76.07569	-2.5
Taurine	Urine	1.1	↘	<0.001	7.8	126.02194	-0.5

**Metabolite**	**Matrix**	**VIP**	**Change^‡^**	***P*-value**	**Chemical shifts (ppm)**		

NMR						
Acetic acid	Rumen	4.2	↘	N/A^∗^	1.93		
Suberic acid	Rumen	2.6	↗	N/A	1.30/1.55/2.16		
C-Alanine	Rumen	1.5	↗	N/A	1.49/3.80		
Butyric acid	Rumen	1.4	↘	N/A	0.90/1.56/2.16		
p-Hydroxyphenylacetic acid	Rumen	1.3	↗	N/A	3.46/6.88/7.17		
Isobutyric acid	Rumen	1.0	↗	N/A	1.06/2.39		
Malic acid	Rumen	0.9	↗	N/A	2.39/2.67/4.31		
Phenylacetic acid	Rumen	0.9	↗	N/A	3.54/7.30/7.39		
	
Phenylacetylglycine	Urine	1.8	↗	N/A	3.68/3.75/7.35/7.40		
Citrate	Urine	1.6	↘	N/A	2.51/2.68		
p-Hydroxyphenylacetic acid	Urine	1.6	↗	N/A	4.02		
Hippurate	Urine	1.5	↗	N/A	3.95/7.54/7.62/7.82		
Ureidopropionic acid	Urine	1.5	↗	N/A	2.39/3.32		
Acetate	Urine	1.4	↗	N/A	1.93		
Betaine	Urine	1.4	↗	N/A	3.26/3.91		
Lactate	Urine	1.4	↗	N/A	1.34/4.14		
Pimelic acid	Urine	1.4	↗	N/A	1.29/1.54/2.17		
Choline	Urine	1.2	↗	N/A	3.52		
Isovalerylglycine	Urine	1.2	↘	N/A	0.94/2.00/2.17		
Trimethylamine	Urine	1.2	↗	N/A	3.28		
Creatine	Urine	0.8	↘	N/A	3.03/3.91		

*^‡^Change in treated compared to non-treated. ^∗^N/A, not applicable because the signals for each molecule are split in several small fractions. ^†^Also named 4-Trimethylammoniobutanoic acid.*

**FIGURE 4:**
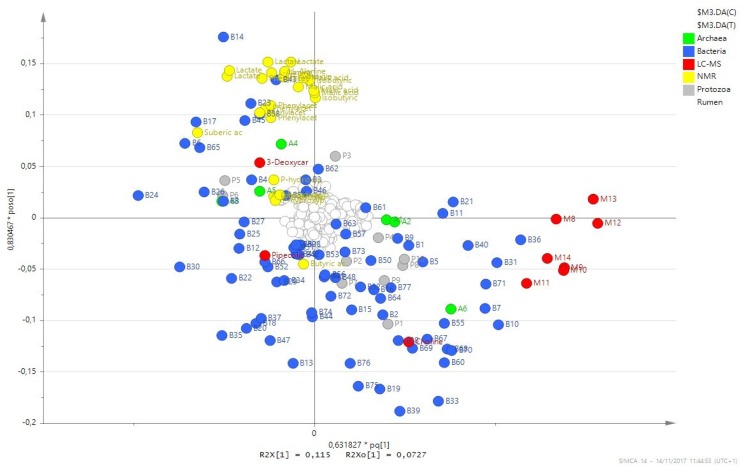
Combined loading plot of the OPLS-DA analysis obtained from rumen fluid samples. Dots represent rumen metabolites detected by NMR (yellow), LC-MS (red-discriminant, white-non-discriminant), archaeal clades (green), bacterial families (blue) and protozoal genera (gray). On the right hand side, metabolites M8 to M14, Bacteroidales B7 and B10, Fibrobacteriaceae B31, Eubacteriaceae B36, an unidentified family from the class Mollicutes B71 and Methanomassiliicoccaceae A6 contributed to the separation between groups.

We performed complementary analyses to gain a better understanding of the relationship between metabolites and microbial groups. A multiblock OPLS-DA analysis, using rumen metabolites determined by LC-MS and NMR and microbial groups (archaeal clades, bacterial families and protozoal genera), shows that the group of seven unknown metabolites with high VIP identified by LC-MS had a high influence in the separation between groups. Microbial groups positioned nearby to these metabolites are the methanogenic archaea Methanomassiliicoccaceae and bacterial families Fibrobacteriaceae, Eubacteriaceae, families from the order Bacteroidales, and an unidentified family from the class Mollicutes (**Figure 4**). The interdependency between metabolites and microbial groups was also revealed by correlation analysis. Only LC-MS was used in this analysis because NMR data consist of signals with different multiplicity, sliced into several buckets, which makes it difficult to calculate the treated/non-treated ratio. Fibrobacteres and other less abundant bacterial phyla, notably Cyanobacteria, Saccharibacteria, Synergistetes, and Tenericutes, were positively or negatively correlated with the majority of VIP metabolites. The archaeal family Methanomassiliicoccaceae was also significantly correlated with most metabolites. In contrast, the main bacterial phyla Bacteroidetes and Firmicutes, other archaea clades and protozoa were poorly correlated with VIP metabolites (Supplementary Figure S4).

## Discussion

This study was designed to test the effect of long-term disturbances on the rumen microbial community of lambs. As defined above, disturbance is an event that can modify the composition of microbial communities, and in this work disturbances were long-lasting events in the form of a dietary additive. The additive was supplied in two periods: during the early rumen microbiota assemblage from birth until lambs had access to adult diets and in established rumen communities later in life. Both the composition and function of the microbial community were monitored at the end of each disturbance period and during the recovery period without treatment. This was done to get insight on questions of microbial ecology that have important implications in health and production of ruminants as it is important to know when modulating the composition and function of the microbiota will be more efficacious.

The indicators of microbial function were methane production and VFA concentration and profile. Treatment consisted in a combination of garlic essential oil and linseed oil. The use of lipids such as linseed oil has been proved effective for reducing methane emissions in fattening bulls and dairy cows (Martin et al., 2016; Doreau et al., 2018). Garlic essential oils have also been shown to reduce methane production although mainly *in vitro* (Mateos et al., 2013; Martinez-Fernandez et al., 2015), as *in vivo* results are more contrasted (Klevenhusen et al., 2011; Ma et al., 2016). The combined feed additive decreased methane production during the time when lambs received the treatment. However, methane emissions recovered to levels similar to control lambs 1 month after the end of the first treatment. In contrast, rumen VFA concentration and profile at 8 weeks of life did not differ between groups, suggesting a different response of the early rumen microbiota to the additive. At 14 weeks no differences in VFA were observed between groups concurring with the absence of differences in methane emissions. However, at 20 weeks of life a decrease in VFA concentration with a greater proportion of propionate and lesser proportion of acetate was observed in treated lambs, suggesting a partial inhibition of rumen fermentation. Nonetheless, lambs’ growth performance was not affected. The absence of persistency on methane emissions is inconsistent with the results of Abecia et al. (2013) that reported decreased methane emissions in goat kids for up to 3 months after the intervention stopped. The treatment was not the same as these authors used a specific inhibitor of the methanogenic pathway (bromochloromethane) and, likewise, the ruminant species differed. Additional studies are needed to better understand these differences. There are a few studies in ruminants reporting the effect of early dietary experiences and microbial inoculation on other phenotypes. Exposure to low quality forages (Distel et al., 1996; Wiedmeier et al., 2002; Xu et al., 2016) and inoculation with adult microbiota (De Barbieri et al., 2015b) in early life improved DM intake and production in animals when adult but the role of the microbiota on these effects was not elucidated. Additionally, in other studies the effect on the desired function did not persist (Ben Salem et al., 2005; Lopez et al., 2016). Interestingly, in our study methane emissions were higher, although only numerically, in 20-week-old lambs that experienced the disturbance in early life. This observation may suggest that the early disturbance increased the resilience of the community or even boosted the methanogenesis function, which in this case was an unwanted outcome.

We monitored the composition of main microbial groups in the rumen, namely methanogens, bacteria, and protozoa. The treatment induced changes, particularly in the methanogens and bacterial communities, during the disturbance. This was somehow expected; however, for bacteria, noticeable differences were observed between the two groups of lambs in the period when not disturbance was applied. The disturbance in early life seemed to induce an alternate assemblage of bacterial communities. The differences between control and treated groups were in the overall bacterial community structure (Adonis *P* = 0.001) and in the relative abundance of up to six phyla and eight families. The families contributing more to these differences were Prevotellaceae, Christensenellaceae, Rikenellaceae, and Lachnospiraceae. The persistency of early-life disturbances on bacterial communities beyond the period of intervention was reported by others (Yáñez-Ruiz et al., 2010; De Barbieri et al., 2015a; Lyons et al., 2017). In contrast and similarly to our results, the less diverse methanogens communities seem to fully recover following disturbance (Yáñez-Ruiz et al., 2010; Lyons et al., 2017) except in one report where methanogens were specifically targeted (Abecia et al., 2013). Lyons et al. (2017) also used linseed oil as additive but reported Succinivibrionaceae, Veillonellaceae and Ruminococcaceae as differentially abundant families. This discrepancy could be due to the additive itself (linseed oil vs. linseed oil – garlic essential oil) and the mode of administration among other differences. However, it is also possible that disturbances render communities unstable and higher or lower abundance of some phylotypes following disturbances are stochastic. In accordance with the tenet that microbial communities in youngsters are not stable (Lozupone et al., 2012), we observed that methanogens and bacterial communities in young lambs were more scattered in NMDS plots whereas communities tend to converge as the lambs aged. The disturbance negatively affected the convergence and could be interpreted as a delay in the maturation of the community. Zaneveld et al. (2017) identified large individual differences in microbiotas within cohorts as a sign of stress. This relatively simple visualization parameter should be monitored over time to ascertain if communities following disturbance are stable or still evolving.

During treatment (disturbance) the archaeal community structure differed at 8 weeks between C1 and T1 lambs and at 20 weeks between C2 and T2 lambs. Across both disturbance periods, the Methanomassiliicoccaceae clade was the most affected by the treatment. Conversely, *Methanosphaera*, a methanogen capable to utilize methylated substrates like the Methanomassiliicoccaceae, increased or tended to increase. However, this increase was proportionally modest indicating that niche replacement was not complete. This agrees with Poulsen et al. (2013) who observed that Methanomassiliicoccaceae decreased in the rumen of low methane-emitting cows treated with rapeseed oil. Martinez-Fernandez et al. (2015) reported that organosulphur compounds reduced methane production and affected methanogens community in continuous cultures. They observed a greater abundance of *Methanobrevibacter* and *Methanosphaera* and lower abundance of *Methanomicrobium* in control vessels, which partially agrees with our results. The abundance of methanogens found in our study might seem contradictory, as there was no difference in methanogens’ numbers when methane emissions were reduced at 8 and 20 weeks and, in contrast, at 14 weeks when methane emissions were similar, methanogens abundance was lower in T1 lambs. We had previously reported the absence of correlation between methanogens numbers and methane emissions (Popova et al., 2011, 2017) that was confirmed by others (Tapio et al., 2017). In contrast, methane production is well correlated with archaeal gene expression (Popova et al., 2011, 2017; Shi et al., 2014).

Changes for some bacterial groups during the disturbance can be ascribed to the feed additive used. Fibrobacteres seemed to be directly affected by the treatment with lower relative abundance in treated lambs at 8 and 20 weeks. In dairy cows supplemented with linseed oil, Yang et al. (2009) found decreased counts of cellulolytic bacteria and decreased abundances of *Butyrivibrio fibrisolvens, Ruminococcus albus*, and *Fibrobacter succinogenes* assessed by qPCR. We calculated the differences between control and treated lambs for the most abundant phylotypes (overall relative abundance higher than 1%). *Fibrobacter sp* was reduced by treatment at 8 and 20 weeks but no differences were observed for the genera classified as *Ruminococcus 1, Butyrivibrio 2*, or Ruminococcaceae NK4A214 group. More recently, Abuelfatah et al. (2016) also showed a decrease in *R. flavefaciens* and *F. succinogenes* using qPCR and rumen content of goats supplemented with linseed oil in the diet. In accord with our results, Patra and Yu (2015) observed that abundance of *F. succinogenes* was reduced by garlic oil.

Metabolomics highlighted some interesting interactions between microbes and metabolites underlining some of the changes observed in the composition of microbial communities and the methane-emission phenotype. There was a group of seven molecules found in the rumen that have not been described before. These molecules are probably associated to the same pathway as they share a common m-amino-pyridine skeleton with different mono-, di- or tri-hydroxy-alkyl groups in orto and para positions. At this stage, it is not possible to speculate on their role and origin but their strong correlation with some microbial groups, e.g., Fibrobacteres and Methanomassiliicoccaceae, could guide further research. A salient feature of the analysis were metabolites that are methyl donors or related to methyl metabolism and that can be associated to methylotrophic Methanomassiliicoccaceae. These metabolites found in the rumen and urine were 3-deoxycarnitine (a precursor of carnitine), betaine, and choline, all known to be converted by gastrointestinal microbes into trimethylamine (TMA) and further biotransformed by the host animal in the liver into trimethylamine N-oxide (TMAO) (Wang et al., 2011). Trimethylamine and TMAO were also discriminant (high VIP) in our study. The decrease in methane emissions and the lower abundance of Methanomassiliicoccaceae in treated lambs was concurrent with the increase of most of these methyl donors, suggesting a role in methylotrophic methanogenesis. One exception was choline in the rumen that decreased although it increased in urine. Other unexpected exception was urinary TMAO that decreased in treated lambs in contradiction with the higher values of urinary TMA.

We conclude that the long-term early-life event disturbance applied to lambs induced modifications in the composition of the rumen bacterial community that persisted after the disturbance ceased. Conversely, no changes were observed in methanogenic archaea and protozoal communities. It is not possible to discriminate whether this is due to the treatment applied or to microbial ecology laws associated to the diversity of these communities, i.e., high diversity for the former and lower diversity for the latter. However, there was no persistency of the early-life disturbance on methanogenesis indicating resilience for this function. On the contrary, early-life treated lambs displayed a numerical increase in methane emissions compared to non-treated lambs. This merits to be confirmed as it could mean that early-life priming could induce emergent properties in microbial communities that are opposed to the effect initially sought.

## Author Contributions

CM, MD, HB, MP, and DM contributed to the initial conception of the study. HB, MP, MB, and DM designed the study. CS, UH, ML, HB, MP, and DM obtained, analyzed, and interpreted the data. CS, UH, HB, and DM wrote the manuscript. All authors approved the submitted versions and agree to be accountable for all aspects of the work.

## Conflict of Interest Statement

The authors declare that the research was conducted in the absence of any commercial or financial relationships that could be construed as a potential conflict of interest.

## Footnotes

1 www.R-project.org
