# Supplementary material for: Effectiveness of Interventions to Modulate the Rumen Microbiota Composition and Function in Pre-ruminant and Ruminant Lambs
Source: Front Microbiol. 2018 Jun 18;9:1273. doi: 10.3389/fmicb.2018.01273 (PMC6015893; doi:10.3389/fmicb.2018.01273)
Supplement: Supplementary file 1 [file Data_Sheet_1.docx]

Supplementary Material

Modulation of the rumen microbiota in pre-ruminant and ruminant lambs by plant-derived antimicrobials

C. Saro^1*^, U.M. Hohenester^1^, M. Bernard^2^, M. Lagrée^3,4^, C. Martin^1^, M. Doreau^1^, H. Boudra^1^, M. Popova^1^, D.P. Morgavi^1*^

*** Correspondence:** [cristina.saro-higuera@inra.fr](mailto:cristina.saro-higuera@inra.fr), diego.morgavi@inra.fr

1. **Supplementary Note 1**

**LC-MS/ToF and NMR analytical methods**

*LC-MS analysis*: Rumen fluid, urine and QC samples were thawed at room temperature, a 100-µl aliquot was transferred into a 1.5 ml polypropylene tube and mixed with 300 µl of acetonitrile containing 0.2 % formic acid. The samples were vortex-mixed and centrifuged at 10 000 g for 10 min. Supernatants were transferred into vials and fitted in an autosampler and 5 µl were injected into the LC–MS system. Separation was performed on a 150 × 2.1 mm i.d. HILIC column (Phenomenex, Torrance, CA) fitted with a same type 2 × 2.1 mm guard column, and using a water/acetonitrile (both containing 10 mM ammonium formate) gradient at a flow rate of 0.3 mL/min. The gradient was started at 0% of A, held for 3 min and increased to 20% in 9 min, then to 70% in 3 min. The gradient was maintained for 3 min before it was returned to initial conditions and maintained for 9 min to re-equilibrate the column prior the next injection. The MS system was operated in positive ionization mode with a scan range of 50–800 m/z. The capillary was set to −4.5 kV, the nebulizer was operated at 2.8 bars; the dry gas was set to 9 L/min at a temperature of 200 °C. The capillary exit was set to 90V with skimmer 1 set to 30V. The time of flight (ToF) was calibrated by using lithium formate (ions at m/z 90 and 800). The MS source was cleaned every 2 series of fifty samples. For accurate mass acquisition, a formate-acetate solution was infused during the run at a flow rate of 100 μL·min^−1^ monitoring for positive ion mode.

Identification of discriminant MS data were performed by tandem mass spectrometry (Source parameters were: capillary voltage 4000 V; nebulizer 40.6 psi; dry gas 9 L/min; dry temperature 200°C). Rumen fluid samples had to be concentrated by evaporation due to low ion abundance in the trap. Four hundreds µL of rumen fluid were diluted with 1200 µL ACN, vortexed for 15 s, and centrifuged at 10,000 g for 10 min at 4 °C. After centrifugation, the supernatant was dried in a GeneVac concentrator (Biopharma Technologies France, Lyon, France) (program HPLC) at 30°C. Dried samples were dissolved in 100 µL 90% ACN.

Fragmentation spectra were obtained on an Orbitrap Velos (Thermo, Les Ulis, France) at normalized collision energies (NCE) 25 and 35 in the ion trap (collision-induced dissociation (CID), resonant activation) as well as NCE 40 and 50 in a quadrupole collision cell (Higher-energy collisional dissociation (HCD), non-resonant activation). Based on these first fragmentation experiments, the collision energy was adapted for each ion species to get optimal fragmentation conditions (signal intensity of the precursor ion: 10-40%). Chromatography were executed on an UltiMate 3000 (Dionex, Les Ulis, France), coupled to an Orbitrap Velos, using the same LC conditions as for the LC-MS acquisitions described before. Due to the different HPLC setting, the final elution/washing step was extended by 3 min to 12 min in total. To obtain the exact mass, full scans were acquired at mass resolution 100,000 (FWHM, m/z 400) whereas mass resolution 30,000 (FWHM, m/z 400) was used for fragmentation experiments, both with AGC 1E6.

*1D NMR analysis*: Rumen fluid and urine samples were centrifuged at 10 000 g during 10 minutes, and 200 µL of the supernatant were mixed with 30 µL of 300 mM potassium phosphate buffer prepared in a 90:10 v/v H2O/D2O mixture. This phosphate buffer contained also 4.6 mM TSP-d4 and 6.1 mM sodium azide (NaN3). Urine and phosphate buffer solutions were mixed by the Gilson Nebula Series robot of the Bruker Metabolic Profiler, which was used to perform the NMR experiments. Final concentrations of the NMR samples in potassium phosphate, TSP-d4 and NaN3 were therefore 39 mM, 0.6 mM and 0.8 mM, respectively. One-dimensional (1D) NMR spectra were acquired on a *Bruker* 500 MHz AVANCE III (Wissembourg, France) equipped with a Prodigy TCI 5 mm probe head. All spectra were acquired using the standard 1D NOESY pulse sequence (noesygppr1d). Typical acquisition parameters were as follows: spectral width 20.57 ppm; 64k time domain points; 128 transients; 4 dummy scans; 10 ms NOESY mixing time 10 ms 90° 1H pulse. The water resonance was pre-saturated during the 4 seconds relaxation delay after initial optimization of the offset.

To obtain higher signals for NMR identification, rumen fluid samples (1.5 mL) were lyophilized, dissolved in 1 mL D_2_O and centrifugated at 10,000 g for 10 min at 4°C. Supernatants were dried in a SpeedVac (Thermo, Les Ulis, France) at 10°C. Dried samples were dissolved in 600 µL phosphate buffer (150 mM phosphate, 40 mg NaN3, 80 mg TSP in D_2_O; pH = 7.0).

Urine samples (1.2 mL) were lyophilized, dissolved in 1 mL D_2_O and dried in a SpeedVac (Thermo, Les Ulis, France) at 10°C. Dried urine samples were dissolved in 600 µL phosphate buffer (150 mM phosphate, 40 mg NaN3, 80 mg TSP in D_2_O; pH = 7.0).

All compounds (Acetyl-carnitine, adenosine, carnitine, choline, creatinine, 3-deoxycarnitine, formic acid, glycerophosphocholine, guanine, 4-hydroxy-L-isoleucine, 7-methylguanine, 2-methylguanosine, pipecolic acid, piperidine, and taurine) used as standard for metabolites identification were analytical grade and purchased from Sigma-Aldrich (Lyon, France).

**2 Supplementary Tables and Figures**

**2.1 Supplementary Tables**

Supplementary Table S1. Sequences and amplicon size of the primers used for the HiSeq Illumina sequencing and quantitative PCR.

| **Target** | **Primer name** | **Primer sequence** | **Usage** | **Size of amplicon (nt)** | **Reference** |
| --- | --- | --- | --- | --- | --- |
| Bacteria 16S rRNA | V3_F357  V5_R926 | 5'-CCTACGGGAGGCAGCAG  5'-CCGTCAATTCMTTTRAGT | Sequencing | 570 | Muyzer et al. (1993) |
| Archaea 16S rRNA | Arch349F  Arch806R | 5'-GYGCASCAGKCGMGAAW  5'-GGACTACVSGGGTATCTAAT | Sequencing | 457 | Takai and Horikoshi (2000) |
| Protozoal 18S rRNA | F566Euk  R1200Euk | 5'-CAGCAGCCGCGGTAATTCC  5'-CCCGTGTTGAGTCAAATTAAGC | Sequencing | 660+ variable | Hadziavdic et al. (2014) |
| Archaea mcrA | q mcrA F  q mcrA R | 5’-TTCGGTGGATCDCARAGRGC  5’-GBARGTCGWAWCCGTAGAATCC | qPCR | 140 | Denman et al. (2007) |
| Bacterial 16S rRNA | 520F  799R | 5’-AGCAGCCGCGGTAAT  5’-CAGGGTATCTAATCCTGTT | qPCR | 280 | Edwards et al. (2008) |

Denman, S.E., Tomkins, N.W., and Mcsweeney, C.S. (2007). *FEMS Microbiology Ecology* 62**,** 313-322.

Edwards, J.E., Huws, S.A., Kim, E.J., and Kingston-Smith, A.H. (2008). *FEMS Microbiology Ecology* 63**,** 141-142.

Hadziavdic, K., Lekang, K., Lanzen, A., Jonassen, I., Thompson, E.M., and Troedsson, C. (2014). *PLOS ONE* 9**,** e87624.

Muyzer, G., De Waal, E.C., and Uitterlinden, A.G. (1993). *Applied and Environmental Microbiology* 59**,** 695-700.

Takai, K., and Horikoshi, K. (2000). *Applied and Environmental Microbiology* 66**,** 5066-5072.

Supplementary Table S2. Relative abundance of archaeal clades in the rumen of control lambs (C1) and lambs treated with a linseed-garlic combination (T1) from birth up to 10 weeks of age. At 16 weeks of age four groups were formed and lambs were retreated or not with the same treatment for 4 weeks (C2 and T2)^1^

|  |  | C1 | T1 |  | P-value* |
| --- | --- | --- | --- | --- | --- |
| 8 weeks |  |  |  |  |  |
| *Methanobrevibacter gottschalkii* clade |  | 0.4174 | 0.3472 |  | ns |
| *Methanobrevibacter boviskoreani* clade |  | 0.0000 | 0.0987 |  | <0.0001 |
| *Methanobrevibacter ruminantium* clade |  | 0.2144 | 0.0236 |  | <0.0001 |
| *Methanosphaera* spp |  | 0.0750 | 0.1240 |  | ns |
| Methanomassilicoccaceae |  | 0.1174 | 0.0775 |  | ns |
| Other archaea |  | 0.0141 | 0.0026 |  | 0.0001 |
|  |  | C1 | T1 |  | P-value* |
| 14 weeks |  |  |  |  |  |
| *Methanobrevibacter gottschalkii* clade |  | 0.5326 | 0.5954 |  | ns |
| *Methanobrevibacter boviskoreani* clade |  | 0.0000 | 0.0000 |  | ns |
| *Methanobrevibacter ruminantium* clade |  | 0.1482 | 0.1372 |  | ns |
| *Methanosphaera* spp |  | 0.0696 | 0.0915 |  | ns |
| Methanomassilicoccaceae |  | 0.1285 | 0.1438 |  | ns |
| Other archaea |  | 0.0224 | 0.0091 |  | 0.0002 |
|  | C1C2 | C1T2 | T1C2 | T1T2 | P-value* |
| 20 weeks |  |  |  |  |  |
| *Methanobrevibacter gottschalkii* clade | 0.4732 | 0.5003 | 0.4760 | 0.5521 | ns |
| *Methanobrevibacter boviskoreani* clade | 0.0000 | 0.0000 | 0.0000 | 0.0000 | ns |
| *Methanobrevibacter ruminantium* clade | 0.1793 | 0.1608 | 0.1274 | 0.1183 | ns |
| *Methanosphaera* spp | 0.0764 | 0.0956 | 0.0655 | 0.0998 | ns |
| Methanomassilicoccaceae | 0.2284^ab^ | 0.1410^a^ | 0.3223^b^ | 0.1896^ab^ | 0.0068 |
| Other archaea | 0.0202 | 0.0223 | 0.0131 | 0.0124 | ns |

^1^Values are median, n= 28 for C1, 28 for T1 8 weeks, 26 for T1 14 weeks, 14 for C1-C2, C1-T2, T1-C2, 13 for T1-T2.  ^a,b^: means with different superscript differ P <0.05. *corrected P-values (Benjamini-Hochberg) resulting from the Kruskal-Wallis test. ns: non significant

Supplementary Table S3. Relative abundance of the bacterial phyla of control lambs (C1) and lambs treated with a linseed-garlic combination (T1) from birth up to 10 weeks of age. At 16 weeks of age four groups were formed and lambs were retreated or not with the same treatment for 4 weeks (C2 and T2)^1^

|  |  | C1 | T1 |  | P-value* |
| --- | --- | --- | --- | --- | --- |
| 8 weeks |  |  |  |  |  |
| Actinobacteria |  | 0.0076 | 0.0035 |  | ns |
| Bacteroidetes |  | 0.5217 | 0.4999 |  | ns |
| Chloroflexi |  | 0.0001 | 0.0001 |  | ns |
| Cyanobacteria |  | 0.0001 | 0.0011 |  | 0.0478 |
| Elusimicrobia |  | 0.0000 | 0.0001 |  | ns |
| Fibrobacteres |  | 0.0939 | 0.0273 |  | 0.0007 |
| Firmicutes |  | 0.2659 | 0.3564 |  | 0.0131 |
| Fusobacteria |  | 0.0000 | 0.0000 |  | ns |
| Lentisphaerae |  | 0.0000 | 0.0000 |  | ns |
| Proteobacteria |  | 0.0025 | 0.0049 |  | ns |
| Spirochaetae |  | 0.0180 | 0.0150 |  | ns |
| Synergistetes |  | 0.0002 | 0.0003 |  | 0.0375 |
| Tenericutes |  | 0.0030 | 0.0026 |  | ns |
|  |  | C1 | T1 |  | P-value* |
| 14 weeks |  |  |  |  |  |
| Actinobacteria |  | 0.0043 | 0.0062 |  | ns |
| Bacteroidetes |  | 0.6114 | 0.5350 |  | 0.0009 |
| Chloroflexi |  | 0.0008 | 0.0021 |  | 0.0157 |
| Cyanobacteria |  | 0.0028 | 0.0013 |  | ns |
| Elusimicrobia |  | 0.0002 | 0.0000 |  | ns |
| Fibrobacteres |  | 0.0365 | 0.0329 |  | ns |
| Firmicutes |  | 0.2866 | 0.3775 |  | 0.0006 |
| Fusobacteria |  | 0.0000 | 0.0000 |  | ns |
| Lentisphaerae |  | 0.0001 | 0.0001 |  | ns |
| Proteobacteria |  | 0.0039 | 0.0038 |  | ns |
| Saccharibacteria |  | 0.0001 | 0.0002 |  | 0.0049 |
| Spirochaetae |  | 0.0119 | 0.0217 |  | 0.0136 |
| Synergistetes |  | 0.0003 | 0.0003 |  | ns |
| Tenericutes |  | 0.0018 | 0.0037 |  | 0.0033 |
| unclassified |  | 0.0000 | 0.0000 |  | ns |
|  | C1C2 | C1T2 | T1C2 | T1T2 | P-value* |
| 20 weeks |  |  |  |  |  |
| Actinobacteria | 0.0052 | 0.0022 | 0.0052 | 0.0051 | ns |
| Bacteroidetes | 0.5815^a^ | 0.6226^ab^ | 0.6342^b^ | 0.7200^b^ | 0.0021 |
| Chloroflexi | 0.0006 | 0.0003 | 0.0004 | 0.0002 | ns |
| Cyanobacteria | 0.0065^a^ | 0.0133^b^ | 0.0148^b^ | 0.0245^b^ | 0.0001 |
| Elusimicrobia | 0.0008 | 0.0010 | 0.0010 | 0.0017 | ns |
| Fibrobacteres | 0.0339^b^ | 0.0047^a^ | 0.0324^b^ | 0.0109^a^ | 0.0004 |
| Firmicutes | 0.3544^b^ | 0.3169^ab^ | 0.2573^a^ | 0.2219^a^ | 0.0026 |
| Fusobacteria | 0.0000 | 0.0000 | 0.0000 | 0.0000 | ns |
| Lentisphaerae | 0.0004 | 0.0006 | 0.0005 | 0.0004 | ns |
| Proteobacteria | 0.0077 | 0.0048 | 0.0138 | 0.0106 | ns |
| SHA-109 | 0.0004 | 0.0002 | 0.0002 | 0.0003 | ns |
| Saccharibacteria | 0.0002^b^ | 0.0000^a^ | 0.0001^ab^ | 0.0000^a^ | 0.0016 |
| Spirochaetae | 0.0099 | 0.0054 | 0.0103 | 0.0082 | ns |
| Synergistetes | 0.0010^a^ | 0.0034^b^ | 0.0006^a^ | 0.0017^b^ | 0.0005 |
| Tenericutes | 0.0032 | 0.0014 | 0.0027 | 0.0010 | 0.0177 |
| Unclassified | 0.0001 | 0.0000 | 0.0000 | 0.0000 | ns |

^1^ Values are median, n= 28 for C1, 28 for T1 8 weeks, 26 for T1 14 weeks, 14 for C1-C2, C1-T2, T1-C2, 13 for T1-T2.  ^a,b^: means with different superscript differ P <0.05. * corrected P-values (Benjamini-Hochberg) resulting from the Kruskal-Wallis test. ns: non significant

Supplementary Table S4. Relative abundance of the bacterial families with an abundance higher than 0,1% and with a significant difference between groups in the rumen of control lambs (C1) and lambs treated with a linseed-garlic combination (T1) from birth up to 10 weeks of age. At 16 weeks of age four groups were formed and lambs were retreated or not with the same treatment for 4 weeks (C2 and T2)^1^

|  |  | C1 | T1 |  | P-value* |
| --- | --- | --- | --- | --- | --- |
| 8 weeks |  |  |  |  |  |
| Lachnospiraceae |  | 0.0589 | 0.1135 |  | 0.0028 |
| Ruminococcaceae |  | 0.0525 | 0.0712 |  | 0.0273 |
| Fibrobacteraceae |  | 0.0939 | 0.0273 |  | 0.0007 |
| Rikenellaceae |  | 0.0526 | 0.0238 |  | 0.0045 |
| Veillonellaceae |  | 0.0012 | 0.0019 |  | 0.0049 |
| Clostridiales_vadinBB60_group |  | 0.0021 | 0.0006 |  | 0.0001 |
| Bacteroidetes_VC2_1_Bac22 |  | 0.0041 | 0.0004 |  | 0.0331 |
| Anaeroplasmataceae |  | 0.0010 | 0.0003 |  | 0.0064 |
|  |  | C1 | T1 |  | P-value* |
| 14 weeks |  |  |  |  |  |
| Prevotellaceae |  | 0.3940 | 0.2639 |  | 0.0001 |
| Lachnospiraceae |  | 0.1029 | 0.1346 |  | 0.0099 |
| Ruminococcaceae |  | 0.0564 | 0.0751 |  | 0.0013 |
| Bacteroidetes_VC2_1_Bac22 |  | 0.0176 | 0.0445 |  | 0.0081 |
| Spirochaetaceae |  | 0.0119 | 0.0217 |  | 0.0097 |
| Acidaminococcaceae |  | 0.0039 | 0.0021 |  | 0.0380 |
| Rhodospirillaceae |  | 0.0015 | 0.0007 |  | 0.0371 |
| Anaerolineaceae |  | 0.0008 | 0.0021 |  | 0.0106 |
| Anaeroplasmataceae |  | 0.0003 | 0.0010 |  | 0.0306 |
|  | C1C2 | C1T2 | T1C2 | T1T2 | P-value* |
| 20 weeks |  |  |  |  |  |
| Prevotellaceae | 0.3224^a^ | 0.4951^b^ | 0.4790^b^ | 0.4660^b^ | 0.0092 |
| Lachnospiraceae | 0.1212^b^ | 0.0926^a^ | 0.0792^a^ | 0.0711^a^ | 0.0006 |
| Christensenellaceae | 0.0889^b^ | 0.0382^ab^ | 0.0668^ab^ | 0.0318^a^ | 0.0127 |
| Fibrobacteraceae | 0.0339^b^ | 0.0047^a^ | 0.0324^b^ | 0.0109^a^ | 0.0004 |
| Veillonellaceae | 0.0080^ab^ | 0.0152^b^ | 0.0051^a^ | 0.0076^ab^ | 0.0103 |
| Bacteroidales_RF16_group | 0.0117^b^ | 0.0031^a^ | 0.0132^b^ | 0.0040^a^ | 0.0007 |
| Clostridiales;Family_XIII | 0.0059^b^ | 0.0066^b^ | 0.0025^a^ | 0.0051^b^ | 0.0002 |
| Gastranaerophilales;uncultured_rumen_bacterium | 0.0020^a^ | 0.0038^ab^ | 0.0062^b^ | 0.0069^b^ | 0.0003 |
| Coriobacteriaceae | 0.0052^ab^ | 0.0022^a^ | 0.0052^b^ | 0.0051^b^ | 0.0454 |
| Gastranaerophilales;Ambiguous_taxa | 0.0017^a^ | 0.0049^b^ | 0.0038^b^ | 0.0042^b^ | 0.0080 |
| Gastranaerophilales;unclassified | 0.0006^a^ | 0.0023^bc^ | 0.0022^b^ | 0.0051^c^ | <0.0001 |
| Synergistaceae | 0.0010^ab^ | 0.0034^b^ | 0.0006^a^ | 0.0017^b^ | 0.0005 |

^1^ Values are median, n= 28 for C1, 28 for T1 8 weeks, 26 for T1 14 weeks, 14 for C1-C2, C1-T2, T1-C2, 13 for T1-T2.  ^a,b^: means with different superscript differ P <0.05. * corrected P-values (Benjamini-Hochberg) resulting from the Kruskal-Wallis test. ns: non significant

Supplementary Table S5. Relative abundance of the protozoal genera of control lambs (C1) and lambs treated with a linseed-garlic combination (T1) from birth up to 10 weeks of age. At 16 weeks of age four groups were formed and lambs were retreated or not with the same treatment for 4 weeks (C2 and T2)^1^

|  |  | C1 | T1 |  | P-value* |
| --- | --- | --- | --- | --- | --- |
| 8 weeks |  |  |  |  |  |
| *Dasytricha* |  | 0.0003 | 0.0000 |  | ns |
| *Enoploplastron*_*triloricatum* |  | 0.0000 | 0.0000 |  | ns |
| *Entodinium* |  | 0.6508 | 0.5428 |  | ns |
| *Eremoplastron* |  | 0.0000 | 0.0000 |  | ns |
| *Isotricha* |  | 0.0147 | 0.0001 |  | ns |
| *Ophryoscolex* |  | 0.0141 | 0.2629 |  | 0.0265 |
| *Polyplastron*_*sp*_LDK-2011 |  | 0.0276 | 0.0001 |  | 0.0462 |
| *Polyplastron* |  | 0.0018 | 0.0000 |  | 0.0348 |
| Trichostomatia; unclassified |  | 0.0084 | 0.0057 |  | ns |
| SAR; unclassified |  | 0.0000 | 0.0000 |  | ns |
|  |  | C1 | T1 |  | P-value* |
| 14 weeks |  |  |  |  |  |
| *Dasytricha* |  | 0.0016 | 0.0000 |  | 0.0157 |
| *Enoploplastron_triloricatum* |  | 0.0000 | 0.0000 |  | ns |
| *Entodinium* |  | 0.5651 | 0.5948 |  | ns |
| *Eremoplastron* |  | 0.0001 | 0.0001 |  | ns |
| *Isotricha* |  | 0.0390 | 0.0201 |  | 0.0301 |
| *Ophryoscolex* |  | 0.1733 | 0.2829 |  | ns |
| *Polyplastron*_*sp*_LDK-2011 |  | 0.0001 | 0.0001 |  | ns |
| *Polyplastron* |  | 0.0001 | 0.0001 |  | ns |
| Trichostomatia; unclassified |  | 0.0279 | 0.0262 |  | ns |
| SAR; unclassified |  | 0.0004 | 0.0004 |  | ns |
|  | C1C2 | C1T2 | T1C2 | T1T2 | P-value* |
| 20 weeks |  |  |  |  |  |
| *Dasytricha* | 0.0527 | 0.0336 | 0.0286 | 0.0159 | ns |
| *Enoploplastron_triloricatum* | 0.0000^a^ | 0.0000^a^ | 0.0003^b^ | 0.0001^ab^ | <0.0001 |
| *Entodinium* | 0.4416 | 0.5877 | 0.5110 | 0.4101 | ns |
| *Eremoplastron* | 0.0002^a^ | 0.0003^a^ | 0.0011^b^ | 0.0004^ab^ | 0.0005 |
| *Isotricha* | 0.0283 | 0.0979 | 0.0179 | 0.0347 | ns |
| *Ophryoscolex* | 0.1900 | 0.1074 | 0.1547 | 0.3360 | ns |
| *Polyplastron*_*sp*_LDK-2011 | 0.0329 | 0.0324 | 0.0687 | 0.0254 | ns |
| *Polyplastron* | 0.0718 | 0.0294 | 0.0687 | 0.0320 | ns |
| Trichostomatia; unclassified | 0.0301^a^ | 0.0342^a^ | 0.1180^b^ | 0.0447^ab^ | 0.0088 |
| SAR; unclassified | 0.0012^b^ | 0.0006^a^ | 0.0007^a^ | 0.0003^a^ | 0.0363 |

^1^ Values are median, n= 28 for C1, 28 for T1 8 weeks, 26 for T1 14 weeks, 14 for C1-C2, C1-T2, T1-C2, 13 for T1-T2.  ^a,b^: means with different superscript differ P <0.05. *corrected P-values (Benjamini-Hochberg) resulting from the Kruskal-Wallis test. ns: non significant

**2.2 Supplementary Figures**


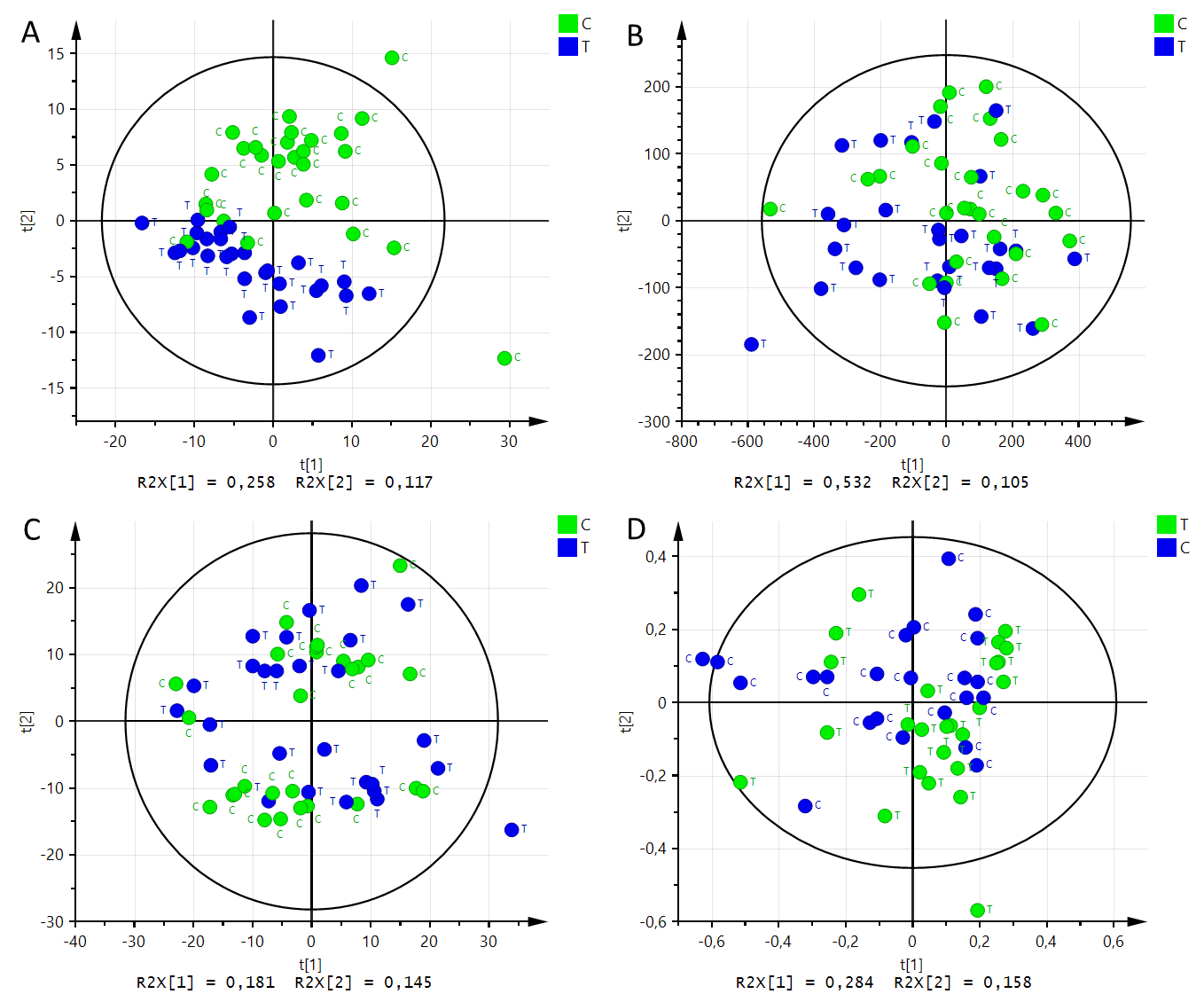
Supplementary Figure S1. PCA of rumen fluid and urine samples from control (C, n= 28 for rumen fluid and n= 27 for urine)) and treated (T, n= 27) lambs at 20 weeks of age. A: rumen fluid analyzed by LC-MS (284 variables). B: rumen fluid analyzed by NMR after variable selection (582 of 641 variables). C: urine analyzed by LC-MS (848 variables); and D: urine analyzed by NMR after variable selection (939 of 1030 variables).


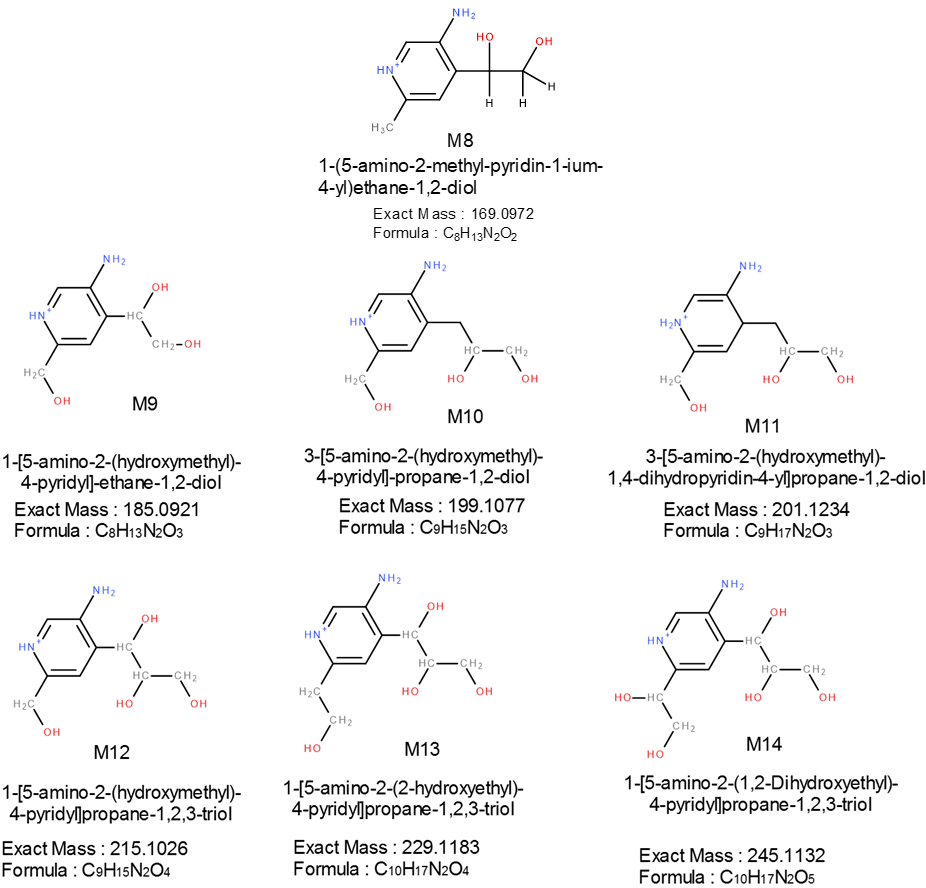


Supplementary Figure S2: Putative structures of the seven unknown molecules found in rumen fluid with their chemical name and formula of the [M+H]^+^ ion.

The putative annotation is based on the specific fragmentation pattern and the similarity between all seven molecules. The high resolution and mass accuracy allow the determination of the elementary formula and therefore allows the use of Ring Double Bound Equations (RDBE). This information restricts the number of possible structures. The release of two H_2_O units suggests that two hydroxyl groups are present in the molecule but the release of H_2_CO indicates an aldehyde group. Both together are not possible because it requires more oxygen than present so a rearrangement during the fragmentation is assumed. To release H_2_CO from a terminal hydroxyl group, a carbocation in β-position is necessary for an 1,2-hydrid shift. The 1,2-hydrid shift leads to a protonated aldehyde, which can be released as H_2_CO. This hypothesis was confirmed by the results of MS^3^ experiments in which the loss of H_2_CO was the second most intense signal after the other hydroxyl groups. The carbocation in β-position were possibly generated by the loss of H_2_O which suggest the presence of a 1,2-diol in the molecule.


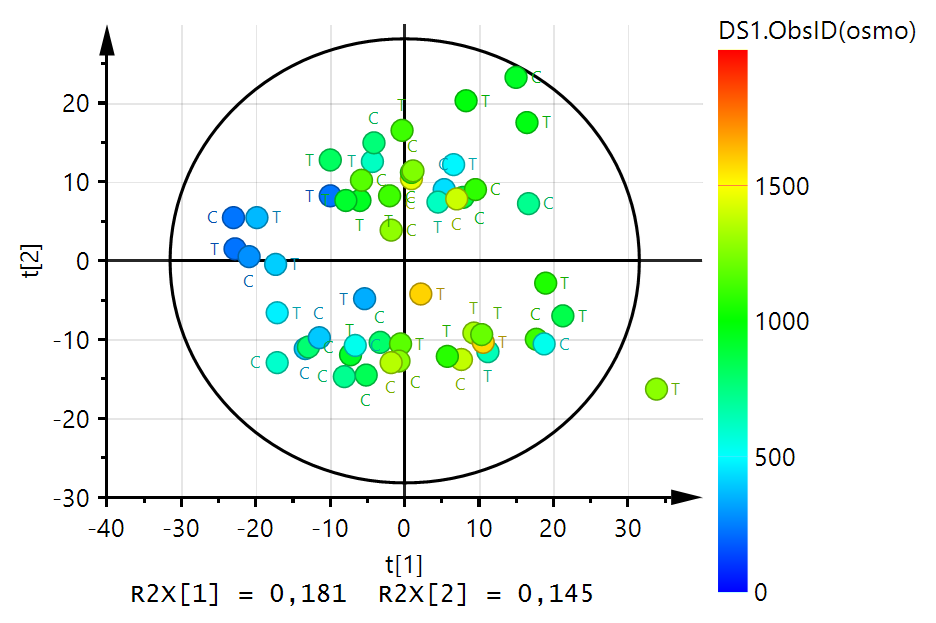


Supplementary Figure S3: PCA plot from the LC-MS analysis of urinary samples colored dependent on their osmolality. Indicating the influence of it on the PCA model.

A)


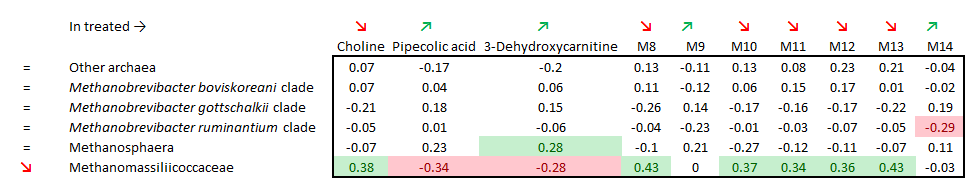


B)


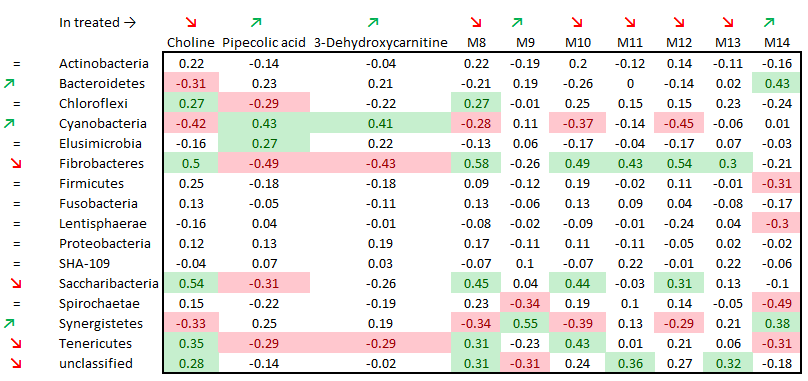


C)


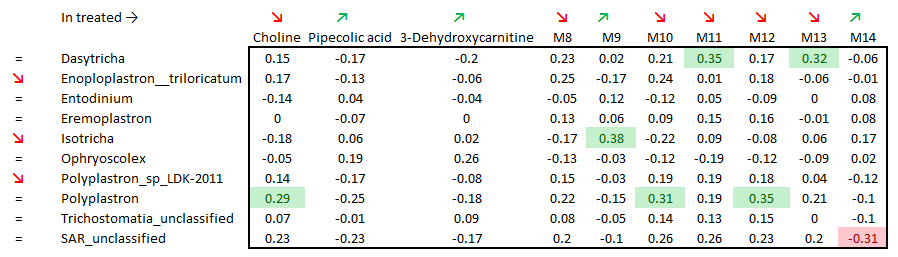


Supplementary Figure S4. Correlation between the relative abundance of A) archaeal clades, B) bacterial phyla and C) protozoal genera and the metabolites found to be discriminant in rumen fluid by LC-MS. Spearman correlation coefficients are highlighted in green (positive) or red (negative) when the correlation was significant. Arrows on top and left edge of the figure indicate if the metabolite or microbial group increased or decreased in treated as compared to control lambs.
